# Designing a prototype trauma registry framework for a tertiary health institution in a low- and middle-income country: A qualitative study

**DOI:** 10.1371/journal.pone.0317141

**Published:** 2025-01-07

**Authors:** Helen Adesoba, Adesola Olumide, Kehinde Oluwadiya, Ajibola Oladiran, Kehinde Ojifinni, Oluwafemi Popoola, Carl Bonander

**Affiliations:** 1 School of Public Health & Community Medicine, Sahlgrenska Academy, University of Gothenburg, Gothenburg, Sweden; 2 Department of Community Medicine, College of Medicine, University of Ibadan, Ibadan, Nigeria; 3 Department of Surgery, Ekiti State University, Ado–Ekiti, Nigeria; 4 Department of Surgery, College of Medicine, University of Ibadan, Ibadan, Nigeria; 5 Emergency Medicine Department, University College Hospital, Ibadan, Nigeria; Duke University Medical Center: Duke University Hospital, UNITED STATES OF AMERICA

## Abstract

**Introduction:**

Low- and middle-income countries experience high injury-related mortality rates, with road traffic crashes being a significant contributor in Nigeria. Data from trauma registries are crucial for designing and advocating for trauma intervention programmes. However, there is limited research to inform the development of trauma registries in a Nigerian setting. The aim of this study was to design a feasible prototype trauma registry (TR) including, scope of activities and registry components for University College Hospital (UCH), Ibadan, Nigeria.

**Methods:**

In-depth interviews were conducted with eight purposively selected trauma registry stakeholders in UCH to obtain context-specific information for a prototype registry. An expert meeting was conducted with four purposively selected experts within the hospital to assess and validate the suitability of the prototype TR scope and TR components, confirming their applicability and potential efficacy in UCH. Information obtained from the interviews and expert meeting were analysed deductively using thematic analysis.

**Results:**

Stakeholders identified the most feasible scope for the trauma registry (TR) as daily data collection on all trauma patients from their initial presentation to discharge or death. This data would be gathered primarily at two critical points: the accident/emergency department and the wards where trauma patients are admitted. Stakeholders believed that comprehensive information about trauma patients could be achieved through these collection points. Following this scope, the analysis led to the identification of 21 essential components and activities for the TR, which were then organised into six categories: registry personnel, computers and other materials, trainings, technology infrastructure, administrative services, and monitoring and evaluation.

**Conclusion:**

The scope and components identified are relevant to our context and have the potential to contribute to trauma prevention programmes, improve patient care and outcomes, and contribute to trauma-related policies and programmes if successfully implemented.

## Introduction

About 12% of the worldwide burden of disease is attributable to injuries [1], while low- and middle-income countries (LMICs) account for over 90% of worldwide injury-related mortality [2]. Several studies have shown trauma to be the primary cause of death and morbidity in emergency departments in Nigeria, with annual injury rate of about 11.2 per 100,000 people [3]. Okereke et al. in a systematic review of injuries focusing on Nigeria, reported that 98.4% of case presentations in the emergency unit in midwestern states were as a result of trauma. In the review, two-thirds of the trauma cases were from road traffic crashes (RTC), while about 5.5% were from distant falls [4]. About one-quarter of fatal cases of RTC have been found to occur in the south-west region of Nigeria, contributing to about 22% of the RTC burden in Nigeria [5, 6].

Gliklich et al. argue that data obtained from a registry created explicitly for the primary purpose of the disease of interest (i.e., primary data sources) have a higher likelihood of being accurate, valid, and reliable because the registry controls the measurement and data collection methods [7]. Many high-income countries have access to relevant and trustworthy data from their trauma registries (TRs) [8]. These data have helped find commonalities in trauma patients’ injury occurrences, access to care, referral channels, and outcomes in the real world. This evidence-based approach has helped make decisions that improve injury care delivery, injury prevention initiatives, and policymaking in many countries. For instance, multiple studies from trauma centres in the USA have demonstrated a reduction in the frequency and severity of head injuries in motorcycle riders who wear helmets as compared to motorcycle riders who do not [9–11]. Moreover, information from TRs has also helped change the minimum legal age required to purchase alcohol, distribute alcohol, and determine the penalties for operating a vehicle while under the influence of alcohol in New Mexico [12]. Furthermore, data from the Dutch TR has proven to be a valuable requirement when evaluating the trauma system’s performance in transferring patients to the right place at the right time [13].

Despite the high burden of injury in Nigeria, injury-related data in Nigeria is mainly recorded in the National Bureau of Statistics (NBS) report, the Federal Road Safety Corps (FRSC) report, and Emergency Department Records [14]. While these data sources help provide insights into injury, the information is sometimes not gathered systematically, making them prone to missing data, misinterpretation of data, and data inaccuracy between sites, amongst others, because they are secondary data sources for injury. Therefore, there is an urgent need for a comprehensive TR that can standardise injury data collection, improve documentation, and support more informed injury prevention strategies and treatment policies.

Developing original trauma registries involves considerable planning and preparation; success can only be achieved after considering local needs, resources, and barriers from the outset [15, 16]. Crucial steps toward achieving this goal include (i) stakeholder engagement, (ii) recruiting and training dedicated registry staff, (iii) integrating the registry’s routine and objectives into the existing health infrastructure [15, 16]. In low-resource settings such as Nigeria, these steps can pose serious challenges [15].

In Nigeria and the broader African context, previous efforts to establish trauma registries have grappled with sustainability issues stemming from limited long-term financing, staff retention, a high number of data variables, case-finding-related challenges, and other factors [16, 17]. Thus, it is vital to prevent the premature demise of a registry initiative by prioritising low-cost, context-specific planning, which is essential for crafting locally appropriate registries.

Existing research on developing trauma registries in low-resource environments is limited [18], necessitating further exploration into the requirements for implementing effective trauma registries in Nigeria. This study addresses this gap by designing a prototype trauma registry for University College Hospital (UCH), informed by best practices outlined by Gliklich et al. [7]. The primary goal of the TR is to improve trauma documentation, laying the foundation for enhanced trauma care and informed injury prevention strategies and policies. Through interviews with local experts and stakeholders, the study aimed to establish a feasible scope and identify essential elements of the registry, customising it to the specific needs and resources available in the setting.

## Methods and materials

The University College Hospital (UCH), Ibadan, Nigeria, is a leading tertiary health facility in south-west Nigeria with 1229 bed spaces. It also receives patients, including trauma cases, from within and outside of the south-west region of Nigeria.

The study was performed in two phases:

- Individual interviews with trauma and registry stakeholders to determine a feasible TR scope and identify TR components (i.e., activities and resource requirements).
- An expert meeting with local stakeholders to assess and validate the TR scope and components.

### Identification of trauma registry scope and components

This study used a qualitative description approach which has been described as suitable for obtaining information that seeks to develop or refine an intervention in a given context [19]. Eight trauma and registry stakeholders (including surgeons, resident doctors, injury prevention experts, consultants who had established TRs in other institutions, and a consultant physician within the hospital who was conversant with the cancer registry operations in the hospital) were purposively selected for the study to obtain broad and rich insights. H.A. (a female public health master’s student at the University of Gothenburg with experience in qualitative research) invited the selected stakeholders via e-mail and telephone calls, provided information about the study, and obtained a verbal informed consent which was recorded.

None of the selected participants refused participation; hence, in-depth interviews were held with the eight trauma registry stakeholders. A semi-structured guide that was developed informed by previous literature on trauma and disease registry development was used for the interviews (S1 File). The interviews were conducted by H.A. virtually through Zoom® platform and WhatsApp audio calls. Probing for more information was done where necessary. While the Zoom® platform allowed access to non-verbal cues, WhatsApp audio calls were used as a substitute for the Zoom® platform where there were network limitations. Respondents remained highly responsive and engaging, irrespective of the platform and all interviews were recorded.

The interviews lasted for an average of 40 minutes. During the interviews, we explored the need for a trauma registry, existing infrastructure, the feasible scope of a trauma registry, components of a trauma registry, funding opportunities, potential challenges, and solutions. These provided crucial insights in determining a feasible scope for a trauma registry. The stakeholders also shared their perspectives on the specific data points, patient demographics, clinical information, and injury severity measures that could be useful in the TR. These were significant in defining the parameters of the registry and ensuring it captured the necessary information for quality trauma care and research. Notes were taken during the interviews, and the audio was transcribed verbatim immediately after each interview.

To increase the rigour in data collection, this study utilised research triangulation of the trustworthiness criteria as described by Lincoln [20] by obtaining information from stakeholders with various experiences and specialties for richer insights. The confirmability and credibility of the data were ascertained by assigning an identification code to all data obtained and quoting participants verbatim for references. In addition, all participants were contacted via email and text messages to review the information they had previously provided for approval and feedback. Only five out of the eight participants responded after multiple attempts to reach out through email and text messages.

The analytical framework used for the study analysis was extracted from the steps to planning a disease registry described by Gliklich et al. [7]. This framework was used to analyse the data deductively to enable a systematic and efficient analysis process. The following themes were identified and named based on what they centered on.

- **Minimum data set (MDS):** These are data elements that mostly capture essential information about the registry goal.
- **Data scope:** This frames the data collection points and frequency of data collection to ensure consistency.
- **Data collection method:** This involves using the best data collection approach, which could be paper-based, electronic-based, or both, since there is no existing data gathering method in place.
- **Registry personnel:** These are those that will be involved in data gathering, collation, analyses, reporting, usage, and ethics.
- **Reporting requirements:** This involves how and how often the registry data should be reported.
- **Database management system:** This is to determine the software application suitable for data collection and management.
- **Training:** These are the steps involved in building new skills and strengthening the existing skills of registry personnel. It involves trainers, designing of a training manual, and determining the mode and length of training.
- **Administrative services:** These are supporting services essential for achieving the registry goal.
- **Sustainability plans:** These are strategies to put in place in order to ensure the continuation and maintenance of the trauma registry. It also helps to achieve the long-term goal of the trauma registry.

Data familiarisation was done to ensure proper understanding, and initial codes were generated from the data by H.A. with critical input from the co-authors. These codes were further assigned to the thematic framework.

### Assessment and validation of TR prototype and registry components

The draft prototype trauma registry (TR) was designed by H.A. based on the findings from the in-depth interviews with trauma and registry stakeholders. The components of the draft TR were determined through these interviews and information from key literature by Gliklich et al. and Purcel et al [7, 21] on the implementation of a hospital-based TR in resource-limited settings and a registry—user’s guide, respectively.

An expert meeting was then conducted with four purposively selected experts within the hospital who are experienced in injury care, injury prevention, hospital data and quality control, and the setting up of disease registries to assess the suitability of the prototype TR scope and components. A written informed consent was obtained from participants via email, and the meeting was held virtually using the Zoom® platform, and the discussion was recorded.

During the meeting which lasted about two hours, the draft TR prototype and the registry components were presented for expert input and validation (i.e., the assessment of the suitability of the TR scope, appropriateness of the TR components, feasibility and sustainability of the TR) based on their knowledge of the hospital structure as well as previous experience establishing trauma databases and pilot registries. Notes and recordings were made during the workshop. The workshop recording was transcribed and analysed based on three indicators: if the participants agreed or disagreed with:

- The scope and components of the presented draft prototype TR.
- Whether the prototype was realistic and could potentially be implemented in the intended UCH context.
- The sustainability of the registry over time.

Information obtained from the expert workshop was used to improve the draft prototype TR.The recruitment of study participants and data collection for this study lasted for 11 weeks, between March 6, 2023, and May 19, 2023.

### Ethical considerations

Ethical approval for this study was obtained from the Oyo State Research Ethical Review Committee, Ministry of Health Secretariat (Ref. No. AD 13 / 479 / 314).

## Results

### Demographic characteristics of in-depth interview participants

A total of eight trauma and registry stakeholders were interviewed, and key sociodemographic characteristics are presented in Table 1.

**Table 1:** Demographic characteristics of in-depth interviews participants.

Participants Characteristics		N (8)	%
Gender	Male	5	62.5
	Female	3	37.5
Specialty	Trauma Surgeon	2	25.0
	Data Access and Manager Expert	2	25.0
	Chief Nursing Officer	1	12.5
	Disease registry user	1	12.5
	Disease registry developer/Epidemiologist	2	25.0

### Findings from the in-depth interviews

The context- specific information for the UCH prototype TR obtained from the in-depth interviews are presented under the following themes with illustrative quotes presented in Table 2.

**Table 2:** Illustrative quotes from in-depth interviews.

Theme	Illustrative quotes
Minimum dataset	“*Selecting a minimum dataset for the trauma registry is important to ensure important data are collected; it has even been shown to contribute to the sustainability of TR*.”—Male, Data Access and Manager Expert
	“*Even though we need a lot of information about patient condition to improve care*, *it might be good to limit the information that will be in the registry for good time and data management*. *Therefore*, *I suggest you consider the WHO dataset for injury as the basis for the minimum dataset and the WHO standardised clinical form*.”—Male, Disease Registry Developer/Epidemiologist
	“*The information that will be obtained from injury victims should be consistent for comparability*. *I think you should use the WHO dataset for injury because it was created to gather information on injury at its minimum*. *It is to be used by existing hospitals*, *and I think it is the easiest*.”—Male, Trauma Surgeon
Data scope	*“Well*, *for a start*, *I think the TR can capture data from the AED while we expand it later*.*”* Male, Trauma Surgeon
	*“I suggest that the TR should at least cover the AED and the hospital ward*. *Even if it will not include the cold cases*, *it should include the ward because patients are meant to be discharged from the AED after 72 hours*, *and appropriate unit continue attending to patients on the ward*.*”–*Female, Chief Nursing Officer
Data Collection Method	*“I have worked in a hospital where electronic medical records are used*, *so to me*, *it is not a big deal to collect trauma data electronically directly*.*” –*Male, Trauma Surgeon
	*“Haa*.. *let us use paper and pen*, *and then later*, *we can enter the data on tablets or computers*. *Do you know why*? *It is easier to trace missing data*. *Also*, *it can be faster because doctors may want that patient case note while the data collector is still waiting for the form to load*.*”* –Female, Data Access and Manager Expert
Registry Personnel	“*…*.*infact*, *for this trauma registry to work effectively*, *you need to employ data clerks that will be entering the data on tablets*, *someone who will conduct routine data quality checks*. *You must ensure the data is secured considering how information is hacked these days*, *where the data will be entered*, *who will have access to the password…”*–Male, Disease Registry Developer/Epidemiologist
	“… *why can’t we use some of those in the medical records*? *We have many of them*, *and I do not think releasing people to assist in gathering trauma data for the hospital should be difficult for the head of the department*.” –Male, Trauma Surgeon* *
	“….. *but now the registry is a separate data that we want to collect*, *okay*, *so a group of people must do that collection*, *okay*? *Are you going to add it to the doctors’ work*? *Which usually will not be successful*. *Do you want to add it to the nurses’ work*? *Which usually will not be successful*, *so one must get people who will now do the extra work*. *The extra work is based on what the doctors have written in the case note*, *which will serve as input for the registry*.”–Male, Disease Registry Developer/Epidemiologist
	“*Since patient data are collected manually*, *entering the trauma data into the database will amount to additional workload which people will be reluctant to undertake unless incentivised*, *or someone is delegated or employed*, *particularly for that purpose*.*” –*Male, Disease Registry Developer/Epidemiologist
Reporting requirements	*“Hmmm…the TR data could be analysed and reported bi-annually or annually*, *it depends on you but*, *it is important to monitor the data early and make necessary adjustments to ensure data accuracy*.*”–*Male, Disease Registry User
	*“It will be good if a governing and oversight committee can be established to monitor the data quality and other related activities; even resident doctors can use the data for their research*, *so it is important*.*”–*Male, Trauma Surgeon
Database management system	“*We can foster a link with the College of Medicine and the trauma unit*, *so that the College of Medicine in their own REDCap can create an account for trauma unit so that we don’t have to create a new REDCap for the TR*”–Male, Disease Registry Developer/Epidemiologist
	“…*stable internet connectivity is necessary for data entry*, *and we all know that the college internet can be messy sometimes*.”–Female, Data Access and Manager Expert
	“*The college internet can be cheaper for the trauma registry staff since they will be registered as a unit*, *but is it reliable*? *One might need an alternative internet source*.*”–*Male, Trauma Surgeon
Trainings	*“The personnel and health workers must be trained because they must understand their tasks well*.*”–*Male, Data Access and Manager Expert
	*“Yes*, *everyone involved in the use and handling of the registry needs to be trained; the registry staff might require additional training because they are at the forefront*. *Even posters can be pasted around the hospital to create awareness for patients and health workers*.*”–*Male, Disease Registry Developer/Epidemiologist
	*“Of course*, *the training manual should be electronic so that staff can find it accessible and to save cost*.”–Male, Trauma Surgeon
	*“Consultants could develop a training manual*, *which will be shared with the registry staff so that they can always refer to it whenever they are confused about how to implement their task*.*”–*Male, Data Access and Manager Expert
	*“But there are halls within the hospital environment where the training can take place*. *You might need to check for the cost*, *but it should not be expensive*.*”–*Female, Chief Nursing Officer
	*“…the registry staff training might take up to 10 half days*, *depending on the content in the training manual*, *but proper training is essential; even refresher training is essential*, *maybe quarterly or bi-annually*.*”–*Female, Data Access and Manager Expert
Administrative services	“*An office space within the hospital is crucial for the TR*. *Also*, *the TR can be situated in the trauma department because it is closer to action*.*”–*Male, Trauma Surgeon
	*“The trauma registry can be situated in the A&E since patients come through that unit*, *but where will the registry staff be sitting*? *Moreover*, *remember*, *the information should be confidential*.*”–*Male, Disease Registry Developer/Epidemiologist
	“*Not every UCH staff has offices talk more of trauma registry staff*, *and you need a place to enter the data*, *or would you like them to enter it in their houses*?*”–*Female, Chief Nursing Officer
Sustainability plans	*“This is a good initiative; we recognise its relevance*, *but what about the maintenance*? *I think the state government can assist with funding it if other health institutions in the state agree*. *Also*, *you might need to cost all items needed; this will give the stakeholders an idea of what is required financially*.*”–*Male, Trauma Surgeon
	*“The hospital or the emergency department should be able to come in*, *though I do not know how funds are allocated to the departments*.*”–*Female, Chief Nursing Officer
	*“This is a good initiative; we could network and secure funds from international donors*, *evaluate the registry data after a year*, *and use that to seek support from the hospital herself; at least we have tried*.*”–*Female, Data Access and Manager Expert

#### Minimum dataset

Since the goal of the TR is to improve trauma data documentation, with the potential for this data to be used to drive injury prevention initiatives and enhance trauma care, the in-depth interview participants emphasised the importance of specifying a minimum dataset for capturing trauma data from selected service points. They also stated that setting a minimum dataset aid in gathering necessary information, improves consistency and accuracy, and prevents undue wastage of time and effort that would otherwise be expended to obtain huge amounts of information that may be difficult to manage over time. The participants believed that specifying a minimum dataset would enhance sustainability of the TR and promote standardisation of information obtained to aid comparability and ultimately ease of use of the data obtained.

#### Data scope

Participants mentioned that trauma data can be captured from different hospital departments depending on the trauma registry’s scope. The data sources mentioned were the general outpatient department, accident/emergency department (AED), mortuary, and hospital wards. More than half of the participants mentioned the AED as crucial for trauma data because new trauma cases come to the hospital through the AED. Some participants said that the TR can capture data from the AED alone as a start, others however suggested that data should be captured from the AED and the ward because patients are typically expected to be discharged to the ward after 72 hours.

#### Data collection method

All participants agreed that data should be collected electronically. However, there were varied opinions on how it should be collected, whether electronically from the onset or using a paper-based datasheet first and later transferred to the electronic database. While some participants agreed that the former was a good option, the majority disagreed and underscored the fact that using a paper-based format for collection at the point of obtaining information would make it easier to trace missing data.

#### Registry personnel

The issue of registry personnel was viewed as crucial by the participants, and many of them identified it as challenging. Most participants mentioned that we need to employ people who will extract the data from patient case notes, a senior person who will review the data for accuracy and completeness, and probably analyse the data at intervals, which is crucial for the evaluation of the TR. In addition, one of the participants mentioned the importance of creating a governance and oversight committee to ensure TR accountability. While some participants feel there are existing human resources in UCH, such as doctors, nurses, and record staff, to assist with the TR, the majority had differing views based on previous experiences with data gathering within the hospital. They felt that it was better to allocate this task to dedicated data clerks rather than overburden doctors or nurses who had their job description with this additional task. This was further explored during the expert meeting, specifically in terms of the competencies needed for data analysis, reporting, utilisation, and ethical considerations.

#### Reporting requirements

Some participants agreed that data from the TR should be reported annually, while others feel it should be reported quarterly or biannually for initial proper monitoring of the registry. Also, establishing a governing and oversight committee was suggested as crucial for reviewing data from the TR.

#### Database management system

All participants agreed that the hospital’s IT department can manage the TR database rather than establishing a new one. The Research Electronic Data Capture (REDCap) application was recommended for data management, however, it requires internet connectivity for data uploads. Unstable internet access highlighted as a potential concern and participants advocate for an alternative source of internet to serve as a backup for the internet service currently provided by the hospital.

#### Trainings

All study participants recommended training sessions for everyone who will be involved in data collection and usage, including health workers and the core registry staff. They deemed this essential to ensure a proper understanding of the registry’s objective, dedication to the data gathering process, and to guarantee accurate data entry. One of the participants suggested that the registry staff might require additional training and practical sessions to ensure proper understanding.

Study participants suggested that a training manual should be developed by trauma and registry consultants for trainees depending on their categories, i.e., health workers and registry staff. They emphasised that the training manual is essential to promote standardised and unified methods, which improve accuracy and consistency in data collection, analysis, and reporting.

The suggested mode of training for the registry staff was to be in-person and within the hospital environment. Even though specific number of training days were proposed, participants agreed that the actual number of training days would be dependent on the training manual.

#### Administrative services

Participants agreed that administrative services are critical to good registry planning. It also ensures the registry operates efficiently, supporting its overarching goals and objectives. Administrative support that were mentioned include office space allotment, printing of training.

#### Sustainability plans

Funding emerged as a paramount challenge, with stakeholders underscoring the necessity for a comprehensive budget and sustainable funding strategies to support the TR’s longevity. Addressing these challenges demands detailed cost estimations and strategic fundraising plans for the TR’s sustainability. Stakeholders identified various potential funders, such as the government, the hospital itself, and international organisations.

Following the in-depth interviews, a draft prototype TR was developed. Data on trauma patients presenting in the hospital AED and those on admission in the wards would be obtained by dedicated registry staff. The minimum dataset to be obtained would be guided by the World Health Organisation (WHO) dataset for injury. The initial TR components were identified including dedicated registry personnel, database and support equipment, stable internet, computers, training and administrative support. There was consensus that the prototype was suitable for the initial phase and would be expanded with time. This prototype was subsequently presented to the team of experts.

### Assessment and validation of trauma registry scope and components

The experts reviewed the scope and components of the draft prototype TR for appropriateness for the context, feasibility, and sustainability. The information obtained from the expert workshop were further used to refine the TR scope and components.

**Fig 1:**
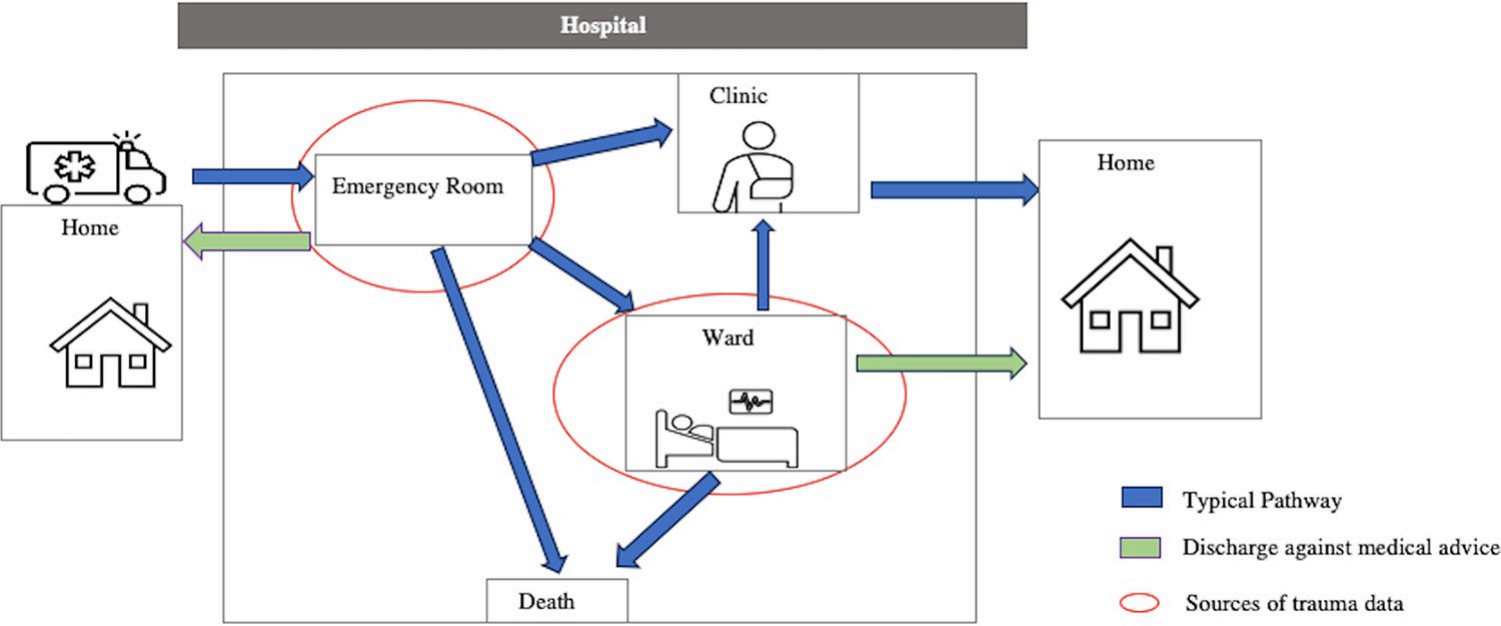
Prototype of a trauma registry for University College Hospital, Ibadan, Nigeria.

The experts unanimously recognised the need to improve existing record-keeping for trauma victims to enhance injury treatment and prevention policies through the development of a trauma registry (TR). They also approved the proposed scope of the TR for University College Hospital (UCH), which involves collecting data from trauma victims in both the accident/emergency department (AED) and wards (Fig 1). The experts emphasised that all injury patients classified according to Chapters XIX and XX of the International Classification of Diseases (ICD-10), as outlined in the WHO Guidelines for Injury Surveillance [22] who present to the AED should be included, regardless of their hospital admission status or injury severity, to ensure comprehensive injury capture. Injury severity will be calculated using the Kampala Trauma Score (KTS), a locally developed tool known for its simplicity and relevance. Their agreement on the data collection points was largely shaped by the limited resources available, although there is optimism for gradual expansion over time. The WHO injury dataset was selected to guide the establishment of the minimum dataset for data collection. After six months of data collection, the experts recommended evaluating the TR to determine if it should focus on specific cases, following a thorough analysis. The estimated annual patient volume is 4,000–5,000 cases, with exact monthly or yearly enrolments to be determined based on initial data analysis.

“*If we are trying to limit the cost, it is better to focus on the outcome in the accident/emergency and the ward, we can decide to expand the scope later in the future.”–Expert -001B*

“*Looking at outcomes from all expected data points gives a robust and holistic view*, *but is it affordable right now*, *is it our priority right now in our own developmental setting*? *I suggest we focus on accident/emergency and include outcome from the wards for a deeper perspective”–Expert -003B*

The TR components initially identified were modified during the expert meeting, resulting in a total of twenty-one components.

Regarding registry personnel, the experts outlined the educational qualifications required for various roles based on their responsibilities and determined the appropriate number of staff. For instance, they agreed that the registry manager should possess strong statistical competence, as this individual will coordinate data analysis. Additionally, the governing and oversight committee must consist of senior consultants at UCH, given their critical roles in overseeing data usage, setting policies, and addressing ethical and legal matters. In addition, they set-up the data collection frequency to be daily to ensure timely and comprehensive information gathering. The experts agreed on training of the registry staff and healthcare workers. However, they provided additional information on the frequency of training. Monitoring and evaluation was included by the experts as part of the TR components to enable continuous monitoring of the project’s progress, track successes, and make necessary adjustments for improvement.

“*As you have mentioned, data collection should be done daily. In addition to that, the ward rotation of resident doctors and house officers should be factored into the periodic training of health workers to ensure that everyone is on the same page.”*–*Expert -003B*

The experts agreed that the prototype TR is feasible and potentially sustainable. Several factors were highlighted as contributing to this conclusion, including having dedicated TR staff, the involvement of healthcare workers, and robust monitoring and evaluation procedures. Funding was identified as a crucial contributor to the TR’s sustainability. The expert suggested that the components be properly costed to provide invaluable insights into the financial implications of implementing the project; this will help to make informed decisions on the development of the TR in UCH.

“*The prototype TR is actually feasible. If I may, I will suggest that the identified components should be costed. This will achieve two things: i) to identify feasible and potential funders ii) to have a cost estimate that can be presented to potential funders”–Expert -002B*

“*While we work on the cost of developing the registry*, *let’s ensure that the core registry personnel are paid while registry personnel such as the governance and oversight committee are provided with modest compensation for the time they spend on meetings to ensure commitment and accountability*, *even though they are UCH staff*.*”–Expert –004B*

The twenty-one components with activities that were agreed as adequate for the functionality of the UCH- TR scope was further divided into six categories. These include registry personnel, computers and other materials, trainings, technology infrastructure, administrative services, and monitoring and evaluation (Table 3).

**Table 3:** Description of prototype trauma registry components and activities.

	Registry components/activities	Total unit	Qualification	Description
	Personnel			
*1*	*Registry Manager*	1	A master’s degree in Demography and social statistics or Public Health (Statistics)	To guarantee data quality and conduct quality assurance and data validation and ensure data extractor officers and data extractor manager are properly trained and supervised. The registry manager also performs data analysis for reporting and database maintenance.
*2*	*Data Extractor Manager*	1	A bachelor’s degree in life sciences or any health-related field.	To ensure data quality assurance and to train, develop, and retain a team of knowledgeable data extractor officers.
*3*	*Data Extractor Officers*	4	Ordinary National Diploma in health sciences	To identify TR cases in the emergency room and on the wards, and to collect additional data during the patient’s hospital stay.
*4*	*Governance and oversight committee*	5 people/4 times per year	Senior consultants involved in trauma treatment, prevention, and policy working within the hospital	To ensure the TR is producing the desired outcomes and is following all applicable policies, laws, regulations, and ethical standards. They will function in the areas of executive or steering, scientific, adjudication, data access, use, and publications, and liaison.
	Computers and Other Material			
*5*	*Desktop computers*	2		
*6*	*Tablets*	4		
*7*	*Development of TR protocol*	2	A senior consultant and a junior consultant involved in trauma treatment, prevention, and policy working within the hospital	To describe the TR background and justification, as well as the study design
*8*	*Development of training manual*	4	Senior consultants involved in trauma treatment, prevention, and policy working within the hospital	To be developed for initial training of TR staff and healthcare workers. This training manual will also be used for recurrent training.
*9*	*Development of information*, *education and communication materials*	2	Consultants involved in trauma care, control, and prevention	To increase awareness of the importance of the TR, deliver public health messages related to TR, trauma prevention and patient care
	Trainings			
*10*	*Training of healthcare workers*	4 working days	Senior consultants involved in trauma care, control, and prevention	Health care workers in various departments (this will include representatives of doctors, nurses, medical records, and other members of hospital management) who will be using the TR will be educated on data privacy, data use, and ethics before running the registry.
*11*	*Initial Training of Registry Staff*	10 working days	Senior consultants involved in trauma care, control, and prevention	Registry staff will be trained on • collecting data from patients’ clinical notes and records. • interacting with the clinical staff to bring their attentions to data that are yet to be collected.
*12*	*Refreshers Training of Registry Staff*	2days/2 per year	Consultants involved in trauma care, control, and prevention	To ensure data quality and that every registry staff is up to date
*13*	*Refreshers Training of healthcare workers*	2 days/ once per year	Consultants involved in trauma care, control, and prevention	To ensure that all staff (but the old and new joiners) are aware and reminded of data privacy, data use, and ethics of the registry
	Technology Software:			
*14*	*Building questionnaire on REDCap*	-		The I.T department of the UCH already have access to REDCap. REDCap is fast and flexible with multi-site access and reliable for data tracking, manipulation, and monitoring user activity.
*15*	*Internet*	6 devices		To improve performance and speed up work processes.
*16*	*Alternate internet*	6 devices		To sustain the speed of work process in case of internet fluctuations and break down in connectivity
		
	Administrative services			
*17*	*Office*, *Facility*, *and Maintenance*	-		An office space will be rented within or around the hospital premises to ensure easy access to departments where data will be collected, to increase collaboration, to foster collective creativity, productivity, and wellbeing.
*18*	*Paper and Registry Copies*	Per month		The data of trauma patients will first be recorded on a paper questionnaire before entering the database. This is for easy tracing of errors in cases of missing data and where data quality is compromised.
*19*	*IEC materials printing*	A5 DI printing full color		The IEC content developed by trauma experts will be printed and distributed across various departments that will be using TR.
*20*	*Office Supplies (Pens*, *Binders*, *Batteries)*	Per month		
*21*	*Monitoring and Evaluation*	10% of overall project.		Roberton and Sawadogo-Lewis (2022) emphasised the reasons for committing to M&E, which include continuous monitoring of projects to track successes and adjust as necessary and periodic reporting and dissemination of project impact.

## Discussion

In this study, we investigated the feasible scope, components, and activities required to develop and sustain a prototype trauma registry at the University College Hospital (UCH) Ibadan by considering available resources and engaging local stakeholders and experts.

The agreed-upon data scope for UCH’s trauma registry (TR) involves collecting data from all trauma victims presenting to the Accident/Emergency Department (AED) and those discharged to hospital wards (Fig 1). This approach is more comprehensive compared to some existing trauma registries in low- and middle-income countries (LMICs). For instance, the national TR in Cameroon, the Karachi Trauma Registry in Pakistan, and the Kamuzu Central Hospital Trauma Registry in Malawi primarily focus on AED data [21, 23, 24].The stakeholders believed that these data collection points would provide quite robust information that could potentially improve trauma care and promote trauma prevention programmes and policies, considering the available resources.

The experts also agreed that the WHO dataset for injury should guide the minimum dataset to be obtained. The WHO injury dataset has been widely adopted in various global contexts, including in African countries like Cameroon and Ethiopia [25, 26], where it is used in their national trauma registries. By adopting this standardised dataset, UCH’s trauma registry (TR) data would have the potential to be comparable with these existing systems. This comparability could facilitate cross-national comparisons and benchmarking, enabling a deeper understanding of injury trends and care outcomes across different settings. Such alignment could lead to valuable insights that drive improvements in trauma care and prevention strategies. Moreover, it positions UCH to contribute to broader regional and global efforts in trauma surveillance and policy development, further strengthening its role in enhancing public health interventions and injury prevention programs.

Challenges to sustainability identified included having sufficient personnel in place that can work on the TR as well as securing a good funding model. These challenges resonate with previous studies which emphasised similar issues faced in TR development and implementation in Nigeria and other LMICs [21, 27, 28]. To address these challenges, UCH stakeholders have proposed recruiting core TR staff and providing modest compensation to part-time registry staff, such as governing and oversight committee members, to increase motivation and commitment. Additionally, potential funding strategies involving the state government, the hospital itself, and international organisations were suggested to mitigate funding challenges.

As part of our future directions, we plan to conduct a cost estimation for the development of the trauma registry (TR) and provide both the design and cost analysis to the hospital management for potential initiation as suggested by the stakeholders. This will provide accurate understanding of the financial implications of implementing and sustaining the TR. It will also enable proper planning and facilitate the allocation of resources.

### Strengths/limitations

Collaborating with local stakeholders offered diverse perspectives and insights, enriching the understanding of the hospital’s operational landscape. Additionally, it justifies that the design process and identified registry components are transferable to a similar context in LMIC. Moreover, deductively analysing the study data using a predetermined framework enabled the systematic identification of themes and patterns relevant to the study objectives, enhancing the rigour and reliability of the analysis.

Despite the representation of diverse perspectives, two limitations include the purposeful selection of respondents which may introduce selection bias, as well as, the limited sample size of respondents which may limit the generalisability of the findings. Additionally, deductively analysing the study data using a predetermined framework may restrict the exploration of emerging themes or relevant insights that do not fit within the framework. The TR designed for UCH is limited to following patients presented at the AED until discharge from the ward and not discharged from the hospital, hence limiting the data gathered with the TR. It also excludes cases that comes through the general outpatient department, limiting the data gathered with the TR.

## Conclusions

Collaborating with stakeholders in the designing of a prototype TR is an essential effort with enormous potential for enhancing her trauma care, research, and patient outcomes. It also provides essential information for improving injury prevention programmes and policies.

## Supporting information

S1 File Guiding questions for the in-depth interviews. (DOCX)
