# Supplementary material for: Designing a prototype trauma registry framework for a tertiary health institution in a low- and middle-income country: A qualitative study
Source: PLoS One. 2025 Jan 7;20(1):e0317141. doi: 10.1371/journal.pone.0317141 (PMC11706365; doi:10.1371/journal.pone.0317141)
Supplement: S1 File — (DOCX) [file pone.0317141.s001.docx]

In-depth interview guide

What is your experience with disease registry systems (clinician, researcher, supervisor, registry initiator, etc.)?

Do you think there is a need for a trauma registry (TR) in health institutions or UCH? Why/why not?

i) Minimum Data Set (MDS):

- What essential data elements should be included in the trauma registry to ensure comprehensive documentation of trauma cases?
- Are there any specific data points that are particularly critical for informing decision-making or improving patient outcomes?

ii) Data Scope:

- What types of trauma cases should the registry encompass? (e.g., all trauma cases, specific types of trauma, severity levels)
- Are there any specific inclusion or exclusion criteria that should be considered when defining the scope of the registry?

iii) Data Collection Method:

- What data collection methods or sources should be utilized to capture trauma-related information accurately? (e.g., patient hospital card, manual collection)
- How frequently should data be collected and updated within the registry?

iv) Registry Personnel:

- What roles and responsibilities should be assigned to personnel involved in managing the trauma registry?
- What qualifications and training should be required for registry personnel?

v) Reporting Requirements:

- What are the reporting requirements for the trauma registry? (e.g., routine reports, ad-hoc analyses, performance indicators)
- Who are the key stakeholders or decision-makers who will rely on the reports generated from the registry?

vi) Database Management System:

- What features and functionalities are essential in the database management system (DBMS) for the trauma registry? (e.g., data entry, validation checks, reporting capabilities)
- Are there any specific requirements or preferences regarding the choice of DBMS?

vii) Training:

- What training programs or resources should be provided to registry personnel to ensure proficiency in data collection, management, and analysis?
- How should ongoing training and professional development be incorporated into the registry's implementation plan?

viii) Administrative Service:

- What administrative support and resources are necessary to facilitate the smooth operation of the trauma registry? (e.g., budget allocation, IT infrastructure, administrative staff)
- How can administrative processes be streamlined to minimize bureaucratic barriers to registry implementation?

ix) Sustainability Plans:

- Do you think UCH has enough resources for the TR?
- What available local resources do you think the UCH TR can leverage?
- What resources do you think can be mobilized?
- From previous experiences, what are the facilitators and challenges of establishing and maintaining a disease registry or other database?
- What strategies should be implemented to ensure the long-term sustainability of the trauma registry? (e.g., funding sources, stakeholder engagement, institutional support)
- How can potential challenges or barriers to sustainability be addressed proactively?
